# Bayesian inference supports the host selection hypothesis in explaining adaptive host specificity by European bitterling

**DOI:** 10.1007/s00442-016-3780-5

**Published:** 2016-11-25

**Authors:** Carl Smith

**Affiliations:** 1 School of Biology, University of St Andrews, St Andrews, UK; 2 Institute of Vertebrate Biology, Academy of Sciences of the Czech Republic, Brno, Czech Republic; 3 Bell-Pettigrew Museum of Natural History, University of St Andrews, Bute Building, St Andrews, Fife, KY16 9TS UK

**Keywords:** Brood parasite, Host–parasite co-evolution, Oviposition, Spawning site, Superparasitism

## Abstract

Generalist parasites have the capacity to infect multiple hosts. The temporal pattern of host specificity by generalist parasites is rarely studied, but is critical to understanding what variables underpin infection and thereby the impact of parasites on host species and the way they impose selection on hosts. Here, the temporal dynamics of infection of four species of freshwater mussel by European bitterling fish (*Rhodeus amarus*) was investigated over three spawning seasons. Bitterling lay their eggs in the gills of freshwater mussels, which suffer reduced growth, oxygen stress, gill damage and elevated mortality as a result of parasitism. The temporal pattern of infection of mussels by European bitterling in multiple populations was examined. Using a Bernoulli Generalized Additive Mixed Model with Bayesian inference it was demonstrated that one mussel species, *Unio pictorum*, was exploited over the entire bitterling spawning season. As the season progressed, bitterling showed a preference for other mussel species, which were inferior hosts. Temporal changes in host use reflected elevated density-dependent mortality in preferred hosts that were already infected. Plasticity in host specificity by bitterling conformed with the predictions of the host selection hypothesis. The relationship between bitterling and their host mussels differs qualitatively from that of avian brood parasites.

## Introduction

The extent to which a parasite exploits different host species, termed host specificity, can vary at a number of levels. Host specificity may vary spatially and temporally, corresponding with morphological, physiological or ecological differences in host and parasite traits or the environments they occupy (Payne 1997; Poulin 2011). Specificity might also reflect previous exposure of hosts to parasitism, parasite prevalence, the availability of intermediate hosts, or the phylogenetic relationships among hosts (Kaltz and Shykoff 1998; Detwiler and Minchella 2009; Poulin 2011; Feeney et al. 2014). Understanding host specificity, both at a proximate and evolutionary scale, is a key question in ecological and evolutionary parasitology and represents a fundamental step in understanding the distribution and spread of parasites in response to ecological change (Poulin et al. 2011). Despite its significance, the factors that shape host specificity are poorly understood, even in well-studied host–parasite systems (e.g., Smith and Myers-Smith 1998; Giorgi et al. 2004; Feeney et al. 2014; Mendlová and Šimková 2014).

In avian brood parasites, host specificity has been the focus of much research. Host preferences have been attributed to variables such as host population size, duration of nesting period, nest type, host aggression, ‘superparasitism’ (repeated parasitism of a host by one species of parasite) and host habitat use (Brooker and Brooker 1990; Payne 1997; Soler et al. 1999; Honza et al. 2002; Patten et al. 2011; Feeney et al. 2014; Soler 2014). Several hypotheses have been invoked to explain host specificity in avian brood parasites (Smith and Myers-Smith 1998). The parasite density hypothesis is a null hypothesis that predicts that host use is simply a function of parasite abundance, irrespective of host species or habitat features (Hoover and Brittingham 1993). The parasite habitat preference hypothesis predicts that the frequency and intensity of parasitism depends primarily on the habitat occupied by hosts (Briskie et al. 1990; Ward and Smith 2000). In contrast, under the host selection hypothesis different host species vary in quality to the parasite. An additional feature of this hypothesis is that high levels of superparasitism could erode host quality, resulting in switches from preferred host species to previously non-preferred hosts. Finally, the host defence hypothesis predicts that parasites avoid hosts that are effective in defending themselves against parasitism. Host defence may vary interspecifically, but also intra-specifically over the host range through geographic variation in evolved responses to parasitism (Briskie et al. 1992). These explanations for host specificity are not mutually exclusive. Thus, a parasite may express both host and habitat preferences simultaneously and be sensitive to variation in host defences.

Bitterling are freshwater fishes that parasitize freshwater mussels as oviposition sites and share many attributes of avian brood parasites (Karplus 2014; Davies 2015; Wootton and Smith 2015). Like avian brood parasites they offer a tractable system for studying coevolution in nature, and have the additional advantage of being amenable to laboratory experiments (Smith et al. 2004). Female bitterling use a long ovipositor to place their eggs in the gill chamber of unionid and margaritiferid mussels (Wiepkema 1961; Smith et al. 2004), and bitterling embryos show a range of highly derived adaptations to enable them to develop in mussel gill chambers (Smith et al. 2004). Female European bitterling (*Rhodeus amarus*) lay multiple small clutches of 2–6 eggs, repeatedly visiting the same or different mussels to oviposit (Reichard et al. 2008; Pateman-Jones et al. 2011). Bitterling embryos remain in their host mussel for approximately one month and impose significant costs on the host by competing for oxygen and nutrients (Spence and Smith 2013), limiting growth and fecundity (Reichard et al. 2006, 2007a) and potentially damaging host gills (Stadnichenko and Stadnichenko 1980). Mussels have evolved defences against bitterling parasitism, primarily by ejecting their eggs and developing embryos (Reichard et al. 2006, 2009, 2012, 2015), but also by rapidly closing their siphons to prevent bitterling oviposition and by diverting the bitterling ovipositor into their mantle cavity rather than their gills (Reichard et al. 2010). Mussel body size appears not to affect bitterling oviposition preference or quality as a host (Smith et al. 2004), though in avian brood parasites this trait is a strong predictor of host specificity (Medina and Langmore 2016). There is good evidence for coevolution between bitterling and host mussels across their respective distributions. For example, host mussels have evolved counteradaptations that enable them to avoid bitterling oviposition, or eject developing bitterling eggs and embryos, while bitterling almost entirely avoid infection by the parasitic glochidia larvae of mussels (Reichard et al. 2006, 2007a, 2010). Notably, these relationships are stronger in regions of ancient bitterling–mussel sympatry compared with regions where the association is more recent (Reichard et al. 2010, 2012, 2015).

The aims of the present study were to investigate temporal changes in host specificity over the course of a spawning season in the European bitterling, a generalist bitterling capable of exploiting a range of hosts, with the goal of establishing which hypothesis for host specificity best fits observed data across years and among populations.

## Materials and methods

### Study sites

Fieldwork was conducted in the southeast of the Czech Republic, at the centre of the natural range of European bitterling in Europe (Van Damme et al. 2007; Zaki et al. 2008; Bryja et al. 2010). Field sites comprised 13 oxbow lakes created during the 1980s situated along a 40-km stretch of the Rivers Morava and Dyje, tributaries of the River Danube. European bitterling and four species of unionid mussel (*Anodonta anatina*, *A. cygnea*, *Unio pictorum* and *U. tumidus*) occur in the region. Oxbow lakes varied in surface area from 6.8 to 22.7 ha. The onset of spawning in bitterling is cued by photoperiod (Shimizu et al. 1994). The bitterling spawning season in these oxbow lakes begins in late April and continues until mid to late June, with a peak in spawning in early May (Smith et al. 2000a). Consequently, for the purposes of this study, a putative start date to the spawning season was designated as the 1st May.

### Embryo releases

The presence of early life stages (hereafter termed embryos for simplicity, but including egg, embryo, larval and juvenile stages) of European bitterling in mussels was estimated in the years 1995–1997 from May to August, with day of sampling scored from 1st May. Rather than dissecting mussels to detect the presence of bitterling embryos, mussels were enclosed in fine mesh bags (mesh size 0.5 × 0.5 mm) measuring approximately 150 × 200 mm and sealed with a Velcro strip. The mesh bags permitted mussels to filter water normally but retained any bitterling embryos that were released. After sealing in a bag, mussels were placed back in the substrate in the exact location from which they had been taken. The water depth at which the mussel was collected was measured to the nearest 10 mm. Bags were checked after 24 h and the number of bitterling embryos that had been released was recorded. Mussels were collected by hand by a diver and were selected as they were encountered. Sampling mussels by hand is an efficient method of collection that provides an accurate picture of the mussel assemblage (Miller and Payne 1993; Hornbach and Deneka 1996; Smith et al. 2000a). To avoid sampling the same mussel repeatedly within lakes within years, mussels were collected from different areas of each lake on each sampling occasion within years. Mussel distribution within lakes shows a marked depth distribution pattern, but no horizontal pattern (Smith et al. 2000a; Smith, unpublished data). Over the 3-year survey 1889 mussels from 13 populations were scored for the presence of bitterling embryos (Table 1). Sampling took place on 54 occasions on 27 discrete dates after 1st May. The distribution of mussels among species reflected the relative frequency of mussels among the oxbow lakes in the study, with 773 *A. anatina*, 430 *A. cygnea*, 371 *U. pictorum* and 315 *U. tumidus* recorded. These data were poorly balanced. Thus, there were no releases of embryos from mussels in August, and data from this month were excluded from subsequent analysis (Table 1). Not all mussel species were encountered in two oxbow lakes, which were also not sampled in every month, and data for these sites were also excluded from the analysis (Table 1). In the resulting subset of data, there were 723 *A. anatina* of which 202 released embryos, 385 *A. cygnea* with 20 showing embryo releases, 339 *U. pictorum* of which 233 released embryos and 285 *U. tumidus* with 82 releasing embryos.

**Table 1:** Number of mussel samples collected during the study from each study lake and in each month

Lake	Month				Total
	May	June	July	August*	
2	36	59	25	0	120
3*	0	0	19	0	19
4	53	63	28	0	144
5	41	22	32	0	95
6	136	82	176	72	466
7	26	54	98	0	178
8	50	37	144	33	264
9	13	58	6	0	77
10	50	20	39	0	109
11*	0	0	33	0	33
12	30	59	65	0	154
13	28	31	33	0	92
14	38	47	53	0	138
Total	501	532	751	105	1889

Data for lakes 3 and 11 and for the month of August (indicated with asterisks) were excluded from the analysis

Handling of the mussels within the mesh bags likely led to the emergence of early stages that would have otherwise remained within the host except in the case of well-developed juveniles that would be expected to depart from the mussel gill once they completed absorption of their yolk-sac. Removing and replacing mussels inevitably resulted in the animal attempting to rebury itself and these movements and contractions of the valves can result in the premature ejection of bitterling embryos.

A subset of data from this survey was previously published in a study by Smith et al. (2000b). In the study by Smith et al. (2000b), only data from nine sites in a single year were considered and only for fully developed juvenile bitterling, not all early life stages, which was the case here. No hypotheses, analyses or findings from Smith et al. (2000b) are repeated in the current study.

### Mussel dissection

Releases of bitterling embryos from mussels served as an indication of the occurrence of spawning by bitterling in a particular mussel. An alternative approach would have been to dissect all mussels collected, but this was considered an unethical approach. However, to establish whether releases reliably reflected the number of developing bitterling in the gills of a mussel, a subsample of 54 mussels was first placed in mesh bags for 24 h in the way described above, then dissected and the number of bitterling embryos counted. Two models were fitted to these data. A Poisson GLM was fitted as follows:

$$P{\text{Released}}_{i} \sim {\text{Poisson}}(\mu_{i})$$

$$E({\text{Released}}_{i} ) = \mu_{i}$$

$${ \log }(\mu_{i} ) = 1.05 + 0.01 \times {\text{Dissected}}_{i} ,$$

where *P*Released_*i*_ is the number of bitterling embryos released from mussel *i* assuming a Poisson distribution with mean *μ*_*i*_. Dissected_*i*_ is the number of embryos dissected from mussel *i*. In addition, a Bernoulli distribution was fitted to the same data as follows:

$$B\text{Released}_{i} \sim {\text{Binomial(}}\pi_{i} )$$

$$E({\text{Released}}_{i} ) = \pi_{i}$$

$${\text{var}}({\text{Released}}_{i} ) = \pi_{i} \times (1 - \pi_{i} )$$

$${\text{logit(}}\pi_{i} ) = - 2.38 + 0.26 \times {\text{Dissected}}_{i} ,$$

where *B*Released_*i*_ is the number of bitterling embryos released from mussel *i* assuming a Bernoulli distribution with mean *π*_*i*_ and variance *π*_*i*_ × (1−*π*_*i*_).

While the presence of embryos in mesh bags was accurately predicted by the number of bitterling early life stages in mussels (Binomial GLM, generalised *R* ^2^ = 0.58), the number of bitterling released was not (Poisson GLM, generalised *R* ^2^ = 0.17). Consequently, embryo releases from mussels were analysed as binomial data, which best reflected the presence or absence of embryos in a mussel gill.

### Statistical analysis

Before applying statistical models a data exploration was undertaken following the protocol described in Ieno and Zuur (2015). The data were examined for outliers in the response and explanatory variables, homogeneity and zero inflation in the response variable, collinearity between explanatory variables and the nature of relationships between the response and explanatory variables. Mussel total length and the depth at which mussels were found were collinear with species and were subsequently dropped from the analysis.

The temporal patterns of infection of mussels by bitterling embryos were modelled using a Bernoulli Generalized Additive Mixed Model (GAMM), which took the following form:

$${\text{Bitterling}}_{ijk} \sim {\text{Binomial(}}\pi_{ijk} )$$

$$E({\text{Bitterling}}_{ijk} ) = \pi_{ijk}$$

$$\eta_{ijk} = \beta + {\text{Species}}_{ijk} + f_{\text{s}} ({\text{Day}}_{ijk} ) + {\text{Oxbow}}_{j} + {\text{Year}}_{k}$$

$${\text{logit (}}\pi_{ijk} ) = \eta_{ijk}$$

$${\text{Oxbow}}_{j} = N(0,\sigma_{\text{Oxbow}}^{2} )$$

$${\text{Year}}_{k} = N(0,\sigma_{\text{Year}}^{2} ),$$

where Bitterling_*ijk*_ is the presence or absence of bitterling parasitism in mussel *i* in oxbow lake *j* in year *k*. Species_*ijk*_ is a categorical covariate with four levels, corresponding with the four species of mussel, while *f*_s_ (Day_*ijk*_) is a smooth function to model non-linear changes in host mussel infection by bitterling over the course of a spawning season. Data exploration showed differences in the temporal pattern of parasitism among mussel species, so a separate smoother was fitted for each species. Model fit with four smoothers, one for each mussel species, gave a better fit than with a single smoother for all species, and so a model with a separate smoother for each mussel species was used. Smoothers were estimated using O’Sullivan splines (Wand and Ormerod 2008). The number of knots per smoother was fixed at five, with knot position permitted to vary. The random intercepts Oxbow_*j*_ and Year_*k*_ were included to introduce a correlation structure between observations for the same oxbow lake and year, respectively.

To make inferences about the parameters in the model a Bayesian approach was used. A Bayesian GAMM is robust in dealing with relatively complex datasets like the one in the present study, specifically unbalanced nested data, dependency due to repeated measures at sampling sites, and a highly varied non-normal response variable (embryo presence). Bayesian models are flexible in allowing the estimation of a posterior distribution of differences between parameters and across levels of factors. These are relatively straightforward procedures using Bayesian inference, but extremely problematic in a frequentist framework (Zuur et al. 2014; Kruschke 2015). Notwithstanding more general reservations in using frequentist analyses (Burnham and Anderson 2014), the probabilities for null hypothesis significance testing are particularly unreliable with mixed models that use smoothing functions (Zuur et al. 2014; Kruschke 2015). In addition, fitting the model in a Bayesian context permitted flexibility in assessing temporal differences in smoothers, and particularly enabled statistically important differences in the incidence of parasitism among host species to be identified across the spawning season, which would be unfeasible in a frequentist setting.

Diffuse or non-informative univariate priors were put on all parameters. The model was fitted in a Bayesian framework using Markov Chain Monte Carlo (MCMC) with the *R2jags* package (Su and Yajima 2012) in the R statistical environment (R Development Core Team 2016) and mirrored the modelling approach outlined for cowbird brood parasitism by Zuur et al. (2014). Three independent Markov chains were run simultaneously with a burn-in of 50,000 iterations and then 500,000 iterations for estimates of parameter and 95% credibility intervals. Chains were thinned every 10th iteration, resulting in 50,000 Markov Chain samples for each estimated parameter. Mixing and autocorrelation of chains were checked visually using trace plots and the Gelman–Rubin statistic (Kruschke 2015). Autocorrelation was low and good mixing was achieved in each case. The Gelman–Rubin statistic was estimated to be less than 1.004 in all cases, indicating good convergence. Model validation showed no evidence of overdispersion, heterogeneity or non-linear patterns in the model residuals (Zuur et al. 2009). As part of the model-fitting process, the model was used to simulate an alternative dataset. This procedure allowed the fitted values to be compared with the simulated data, with probability values for each data point used to assess model fit. A probability of 0.49 indicated the model complied closely with the data (Zuur et al. 2014).

To examine whether there were temporal changes in the relative abundance of host mussels among lakes among years a binomial GLM was fitted to data for the abundance of each host species. The model was fitted as

$${\text{Number}}_{i} \sim {\text{Binomial}}(\pi_{i} , {\text{Total}}_{i} )$$

$$E({\text{Number}}_{i} ) = {\text{Total}}_{i} \times \pi_{i}$$

$${\text{var}}({\text{Number}}_{i} ) = {\text{Total}}_{i} \times \pi_{i} \times (1 - \pi_{i} )$$

$$\eta_{i} = \beta + {\text{Oxbow}}_{i} + {\text{Year}}_{i}$$

$${\text{logit(}}\pi_{i} ) = \eta_{i} ,$$

where Number_*i*_ is the abundance of host species *i* and Total_*i*_ is the total abundance of all other host species. The model was fitted to a subset of lakes for which there were data for all mussel species in all years, which comprised a dataset from four lakes (lakes 2, 6, 8 and 13) over three years (1995–1997).

## Results

**Fig. 1:**
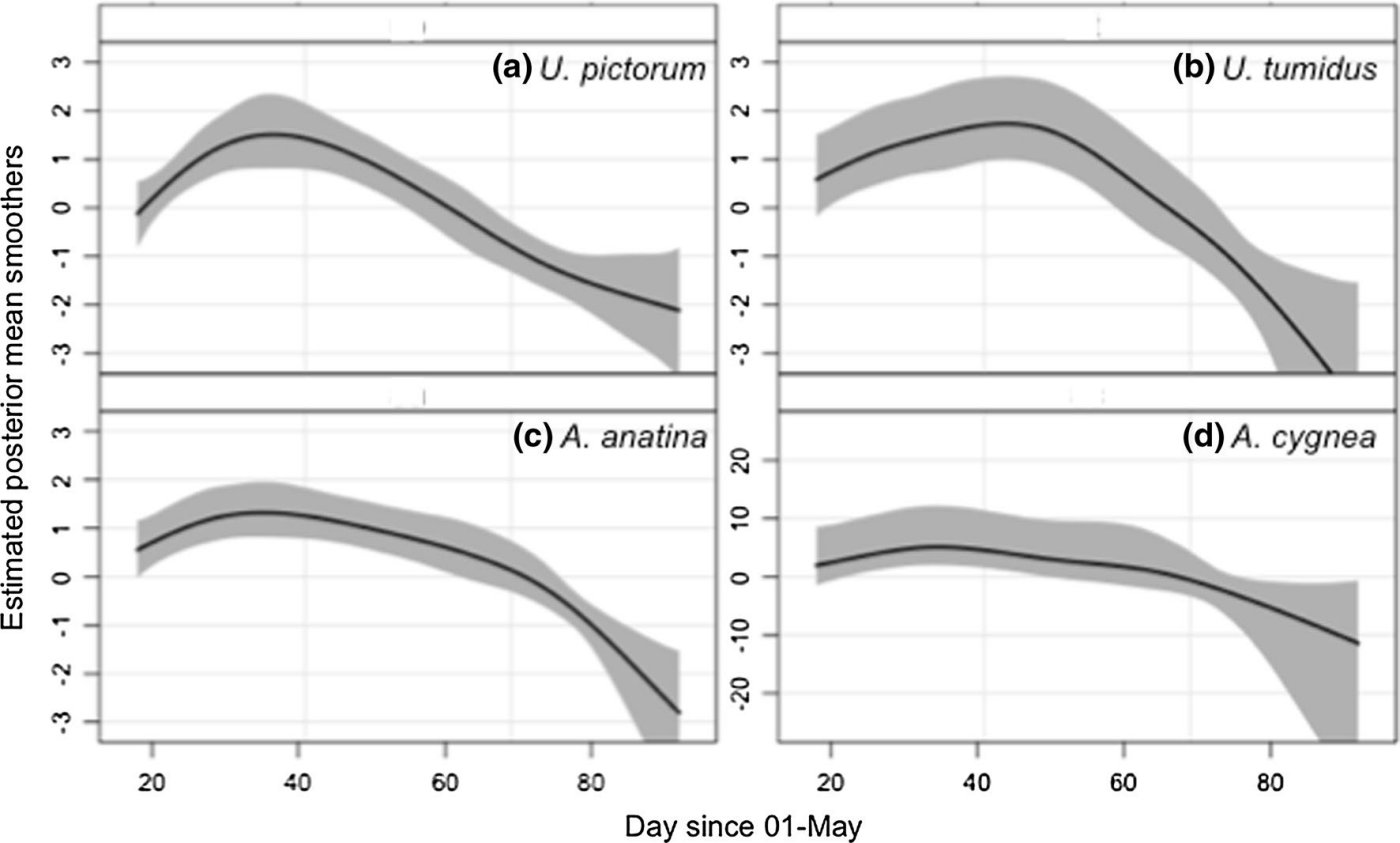
Posterior mean smoothers (*solid line*) and 95% credible intervals (*shaded area*) of European bitterling (*Rhodeus amarus*) parasitism of different host mussel species **a** *Anodonta anatina*; **b** *A. cygnea*; **c** *Unio pictorum*; **d** *U. tumidus*, for day since 1st May (the putative start of the bitterling spawning season) for a Bernoulli GAMM estimated by MCMC and comprising 50,000 Markov Chain samples for each estimated parameter

Parasitism of mussel hosts by bitterling varied across the spawning season, showing a peak at the end of May. Posterior mean smoothers for all species showed non-linear effects with day that deviated from zero (Fig. 1). Prevalence of bitterling early life stages in all host mussel species was greatest between days 35 and 45 (04 Jun to 14 Jun), though the period over which bitterling embryos were encountered varied among species, with embryos recovered from *A. cygnea* between days 18 and 52 (18 May to 21 Jun), while bitterling were recovered from *U. pictorum* from day 18 to 92 (18 May to 31 Jul). Early life stages of bitterling were recovered from *A. anatina* and *U. tumidus* from day 18 (18 May) to 74 (13 Jul).

**Fig. 2:**
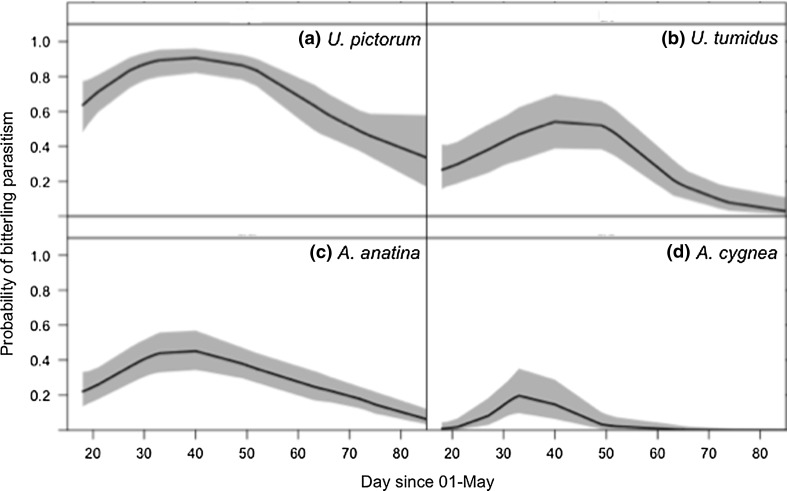
Mean fitted probability (*solid line*) of parasitism by European bitterling (*Rhodeus amarus*) and 95% credible intervals (*shaded area*) for different host mussel species **a** *Anodonta anatina*; **b** *A. cygnea*; **c** *Unio pictorum*; **d** *U. tumidus*, for day since 1st May (the putative start of the bitterling spawning season) for a Bernoulli GAMM estimated by MCMC comprising 50,000 Markov Chain samples for each estimated parameter. Probabilities were derived by adding the intercept, species effect, smoother and covariate, and applying the inverse logistic link function for each MCMC iteration

The temporal pattern of parasitism varied among host mussel species (Table 2). *U. pictorum* was consistently the most parasitized mussel species, followed by *A. anatina* and *U. tumidus*, while *A. cygnea* experienced the lowest probability of parasitism (Fig. 2). Posterior mean probabilities of bitterling parasitism for *U. pictorum* were significantly higher than for *A. anatina* (Table 2). In contrast, the posterior mean probability of parasitism for *A. cygnea* was significantly lower than for *A. anatina* (Table 2), while there was no evidence for a difference in bitterling prevalence between *U. tumidus* and *A. anatina* (Table 2).

**Table 2:** Parameter estimates of mussel infection by European bitterling modelled using a Bernoulli GAMM

Model parameter	Posterior mean	Lower CrI	Upper CrI
Fixed intercept_(*anatina*)_	−1.65	−4.82	1.49
*Species*_(*cygnea*)_	−5.01	−**11.96**	−**2.09**
*Species*_(*pictorum*)_	2.41	**1.91**	**3.02**
*Species*_(*tumidus*)_	−0.01	−0.95	0.75
Fixed intercept_(*cygnea*)_	−6.58	−**11.11**	−**2.46**
*Species*_(*anatina*)_	4.95	**2.32**	**8.44**
*Species*_(*pictorum*)_	7.34	**4.74**	**10.83**
*Species*_(*tumidus*)_	4.89	**2.10**	**8.51**
Fixed intercept_(*pictorum*)_	0.75	−2.24	3.68
*Species*_(*anatina*)_	−2.37	−**2.96**	−**1.91**
*Species*_(*cygnea*)_	−7.65	−**11.47**	−**4.74**
*Species*_(*tumidus*)_	−2.44	−**3.46**	−**1.74**
Fixed intercept_(*tumidus*)_	−1.69	−**5.01**	−**1.68**
*Species*_(*anatina*)_	0.04	−0.70	1.02
*Species*_(*cygnea*)_	−5.47	−**12.65**	−**2.12**
*Species*_(*pictorum*)_	2.43	**1.74**	**3.39**
Random intercept_(*oxbow*)_	0.18	**0.01**	**0.46**
Randon intercept_(*year*)_	1.89	−0.24	0.97

CrI is the 95% Bayesian credible interval

Parameter estimates are presented for each host species as the baseline category

Credible intervals that do not contain zero in bold to indicate statistical importance

**Fig. 3:**
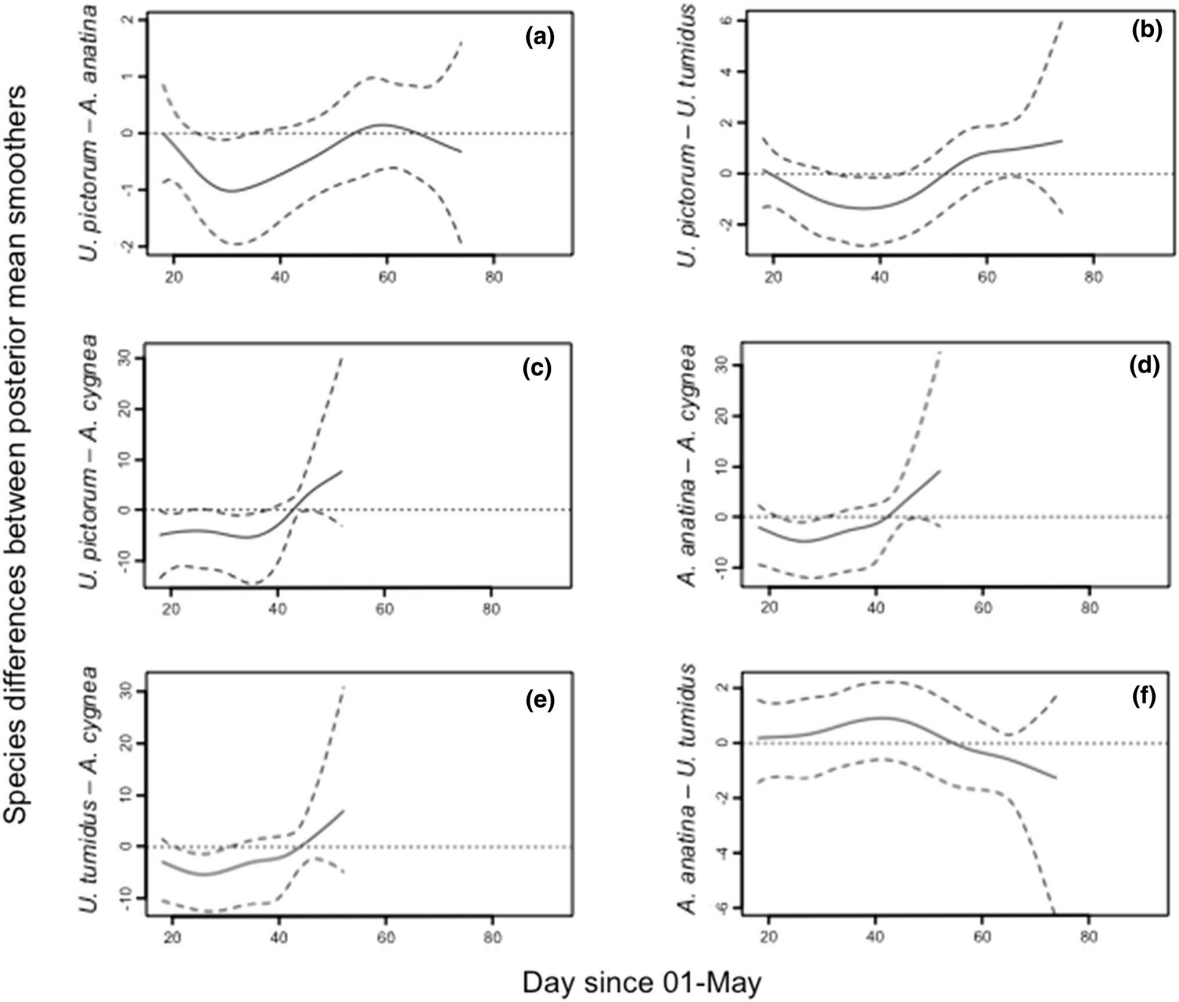
Pairwise differences in parasitism by European bitterling (*Rhodeus amarus*) between 50,000 estimated smoothers for mussel hosts. The *solid line* is the posterior mean of the differences and the *dashed lines* the 95% credible intervals for: **a** *U. pictorum* vs. *A. anatina*; **b** *U. pictorum* vs. *U. tumidus*; **c** *U. pictorum* vs. *A. cygnea*; **d** *A. anatina* vs. *A. cygnea*; **e** *U. tumidus* vs. *A. cygnea*; **f** *A. anatina* vs. *U. tumidus*

Mean differences between the posterior mean smoothers for host mussels identified the periods of the bitterling spawning season when there were changes in the utilisation of host species. *U. pictorum* was significantly preferred over *A. anatina* at the start of the season, but *A. anatina* subsequently showed an increase in infection prevalence during the middle part of the spawning season (days 34–65) (Fig. 3a). At the end of the spawning period, *A. anatina* declined in infection frequency faster than *U. pictorum*. This pattern was repeated between *U. pictorum* and *U. tumidus*, with *U. pictorum* showing a greater increase in bitterling prevalence up until day 34 and with *U. tumidus* subsequently showing a greater increase in infection prevalence between days 53 and 63 (Fig. 3b). In contrast, *U. pictorum* showed a greater increase in infection frequency compared with *A. cygnea* from the onset of data collection until day 38 (Fig. 3c). From day 43 to 52 *U. pictorum* showed a greater decline in bitterling infection than *A. cygnea*. A comparable, but less pronounced, pattern was shown between *A. cygnea* and *A. anatina* (Fig. 3d) and *U. tumidus* (Fig. 3e). *A. anatina* and *U. tumidus* showed no significant difference in the posterior mean of the smoothers (Fig. 3f), indicating no difference in the pattern of preference by bitterling for the two species over the spawning season.

The relative temporal abundance of host mussels was consistent within lakes among years for all mussel species (Table 3).

**Table 3:** Summary of binomial GLM to examine the relative temporal abundance of host mussels within lakes among years for each host species

Parameter	Estimate	S.e.	*z*	*P*
*A. anatina*				
Intercept	−0.316	0.183	−1.73	0.084
Lake × year	−0.001	0.001	−0.58	0.565
*A. cygnea*				
Intercept	−0.447	0.189	−2.36	0.018
Lake × year	−0.001	0.001	−1.29	0.196
*U. pictorum*				
Intercept	−2.202	0.271	−8.13	<0.001
Lake × year	−0.001	0.001	1.19	0.233
*U. tumidus*				
Intercept	−2.204	0.267	−8.27	<0.001
Lake × year	−0.001	0.001	1.44	0.149

## Discussion

The distribution of early life stages of bitterling recovered from four host species of mussel revealed a clear-cut pattern. One species, *U. pictorum*, showed the highest prevalence of infection over the course of the bitterling spawning season while *U. tumidus* and *A. anatina* showed a lower level of infection by bitterling, though they did not differ from each other. A fourth host species, *A. cygnea*, was utilized least. This is the first replicated population-level study to demonstrate this pattern of host specificity by European bitterling.

These changes in host utilization by bitterling reflect adaptive behavioural preferences observed in lab and field experiments. In a previous study (Smith et al. 2000b) it was established that the mortality rate of the early life stages of bitterling during incubation varied among host species. *U. pictorum* proved the best host and *A. cygnea* the worst, with *U. tumidus* and *A. anatina* intermediate between these two. Bitterling spawning preferences reflected this variation in host quality, with female bitterling preferentially ovipositing in *U. pictorum* and avoiding *A. cygnea*. Why these mussels vary in quality as hosts is not wholly clear, but may reflect differences in oxygen conditions inside the mussel gill chamber which favour embryo development (Spence and Smith 2013). Alternatively, or additionally, the gill structure of different mussel species may better suit embryo development (Liu et al. 2006).

While there are clear differences in host quality, which are mirrored by variation in host preferences by bitterling, host quality declines with the density of bitterling embryos in the mussel gill chamber; bitterling embryo survival is negatively density-dependent (Smith et al. 2000b). An outcome is that bitterling reduce the frequency of oviposition in preferred hosts in favour of non-preferred hosts as the former decline in quality through ‘superparasitism’ (sensu van Dijken and Waage 1987). Because increasing embryo density erodes mussel quality, a point can be reached when the preferred host species is comparable in quality to unparasitised individuals of the next preferred species, at which point a shift in host preference is predicted. Using Bayesian inference, plots of posterior mean smoothers in the present analysis permitted the timing of this temporal change in preference to be identified. Thus, *U. pictorum* is shown to be preferred to *A. anatina* and *U. tumidus* early in the spawning season (Fig. 3a, b), with a subsequent increase in the rate of parasitism of *A. anatina* and *U. tumidus* as bitterling began substituting already parasitized *U. pictorum* with the next best alternative hosts. Thus the pattern of posterior mean smoothers is a reflection of the dynamic temporal change in host quality as spawning occurs, but with the order of preferred hosts the same over the spawning season.

The findings of the present study provide support for the *host selection hypothesis*, with the preferred host the one that provides the highest quality oviposition site for bitterling. Erosion of preferred host quality through superparasitism generates temporal changes in host quality that result in temporal changes in host specificity. The primary determinant of host quality that explains the observed host specificity by bitterling has yet to be conclusively identified, but is probably the dissolved oxygen conditions inside the mussel gill (Smith et al. 2001; Spence and Smith 2013; Smith and Reichard 2013). Mussel species vary in their capacity to extract oxygen from water entering their gill cavity (Smith et al. 2001). Notably several cues are used by bitterling in making oviposition decisions, including ophthalmoception, chemoreception and tactioception (Smith et al. 2001, 2004, 2014). However, female bitterling in particular show a strong response to the dissolved oxygen concentration of water emerging from the exhalant siphon of a mussel in making oviposition-site decisions (Smith et al. 2001).

The analysis failed to demonstrate support for the parasite habitat preference hypothesis. A habitat variable, water depth, was measured in the study but was collinear with species and was subsequently dropped from the analysis to (Zuur et al. 2010). If collinearity was ignored, and depth included in the model as a covariate, a significant effect was detected, with greater prevalence of parasitism at shallow depths. However, caution is needed in the interpretation of this result because *A. cygnea* occurred at a greater mean depth than the other three host species. Given that *A. cygnea* was also the overall least preferred host species, this depth effect is most likely driven by the vertical distribution of hosts. If *A. cygnea* was excluded from the analysis the depth effect was not statistically important.

Native unionid mussel populations across much of continental Europe express a limited capacity to eject or avoid bitterling eggs (Reichard et al. 2010, 2012). This situation contrasts with mussel populations in the Pontic region, which show several adaptations to avoid European bitterling parasitism (Reichard et al. 2010, 2015). This difference in response is likely due to the shorter duration of sympatry and lower encounter rate with *R. amarus* in west and central Europe compared with mussels in the Pontic region, where the length of the association may be as much as two million years (Bryja et al. 2010; Reichard et al. 2015). Consequently, the host defence hypothesis does not explain the observed host specificity, though this explanation may apply to host specificity elsewhere in the range of the European bitterling where the bitterling–mussel association is longer established. In Asia, where there are somewhere in the region of 70 bitterling species (Chang et al. 2014; Kawamura et al. 2014), with long historical associations with freshwater mussels there is good evidence for coevolutionary responses between bitterling and host mussels (Liu et al. 2006; Reichard et al. 2007b, 2015; Kitamura et al. 2012), and host responses may play a role in shaping host specificity.

In the case of avian brood parasites, in contrast to the present findings, it is typically the host defence hypothesis that best describes patterns of host specificity (Feeney et al. 2014). There is some evidence for the host selection hypothesis for host specificity in cowbirds (Mason 1986; Smith and Myers-Smith 1998), though this may be an exception (Briskie et al. 1990). However, attempts to quantify host ‘quality’ characteristics, measured in fitness terms, have not been systematically conducted in avian brood parasites. In addition, in cases where selection for improved host defences means that formerly profitable hosts acquire efficient defences, resulting in reduced parasite fitness, formerly unprofitable hosts effectively become profitable, with a predicted switch in parasite host preference (Soler 2014). Thus the unstable dynamics of avian brood parasites, driven by evolving host defences, means that a form of host selection must operate, with the implication that host quality is not a fixed property of a host species. Given the known variation in host responses in the bitterling–mussel system (Reichard et al. 2010, 2012, 2015), the same dynamic process may operate and warrants investigation.

The present data support the observation that the European bitterling is a generalist parasite, able to exploit a range of host mussel species (Smith et al. 2004). A possible explanation for its low host specificity is that host taxa are closely related, with specialisation to exploit one species also permitting exploitation of other species in the same lineage (Poulin 2011). However, evidence from a recent comprehensive phylogenetic analysis of the unionid mussels demonstrated an ancient divergence of the Unioninae (including *U. pictorum* and *U. tumidus*) and Anodontinae (*A. anatina* and *A. cygnea*) (Lopes-Lima et al. 2017). Thus, while European bitterling readily exploit both *U. pictorum* and *U. tumidus*, they also use *A. anatina* but avoid *A. cygnea*, a situation that fails to support a macroevolutionary explanation for observed host preferences. A future approach might examine the ‘functional diversity’ of hosts, based on host species traits (sensu Medina and Langmore 2016), perhaps focusing on the internal environment of the mussel gill as a site of incubation or host habitat preferences.

A caveat to the findings of the study is that vulnerability to premature ejection of bitterling embryos might be host species specific, thereby influencing intra-specific differences in patterns of ejection. However, if the case, a predicted outcome would be a difference in ejection rates between *Anodonta* spp. and *Unio* spp. which differ in gill anatomy (Liu et al. 2006). In reality the ejection of embryos varied as much within genera as between genera. Thus *A. anatina* differed from *A. cygnea*, and *U. pictorum* differed from *U. tumidus*, while *A. anatina* did not differ from *U. tumidus*. The risk of bias from mussel-specific ejection rates was also mitigated by the dissection data, which demonstrated that while ejections did not reliably reflect the number of embryos on the gills of each species, it did reflect presence of embryos, irrespective of mussel species.

The impact of bitterling on host mussels at the population level has yet to be investigated. Inhibiting oviposition by bitterling significantly enhances mussel growth (Reichard et al. 2006). There is strong evidence across several unionid mussel species that mussel size is positively correlated with fecundity (Bauer 1994); thus any reduction in mussel growth will potentially translate into a fitness cost. Further experimental and modelling studies might address the extent to which bitterling regulate unionid mussel populations, a group that is threatened globally (Lopes-Lima et al. 2014).

In conclusion, this study demonstrates a clear temporal shift in host specificity by a generalist parasite of its host species. Changes in host specificity reflect temporal changes in host quality as a result of superparasitism and provide support for the host selection hypothesis in the host preferences of European bitterling.
